# Recommendations for recognizing and diagnosing Acute Hepatic Porphyria in atypical patient populations

**DOI:** 10.1186/s13023-025-04067-7

**Published:** 2025-10-24

**Authors:** Katharina Schmolly, Vivek Rudrapatna, Simon Beaven

**Affiliations:** 1 Present Address: Medical Genetics, University of California Los Angeles, 300 Medical Plaza, Los Angeles, CA 90095 USA; 2 Division of Gastroenterology and Hepatology, University of California, San Francisco, CA 94143 USA; 3 Bakar Computational Health Sciences Institute, University of California, San Francisco, CA 94143 USA; 4 Division of Gastroenterology, University of California, Los Angeles, CA USA; 5 Chair of Gastroenterology, Olive View-UCLA Medical Center, Sylmar, Los Angeles, CA 91342 USA

**Keywords:** Acute Hepatic Porphyria, Underdiagnosis, Rare disease diagnosis, Clinical manifestations, Diagnostic delays

## Abstract

**Background:**

Acute Hepatic Porphyria (AHP) is a group of four rare genetic but treatable diseases that often go undiagnosed due to its non-specific symptoms, under-recognition of the condition by clinicians, and the lack of access to specialists and appropriate testing. This case-control study investigates the phenotypic and demographic patterns in AHP patients at a tertiary care center (University of California Los Angeles) to update recommendations for recognition and diagnosis of this disease in our community.

**Method:**

A retrospective chart analysis was conducted on 45 patients who were evaluated for AHP, Electronic Medical Record (EMR) data was collected and analyzed to investigate clinical differences and correlations.

**Results:**

27 patients tested positive for AHP through urinary metabolites and confirmatory genetic testing and 18 patients tested negative; of those, 16 patients received a definite alternative diagnosis. Hashimoto’s, Type 1 Diabetes Mellitus (T1DM), Fibromyalgia and cannabinoid use with cyclic vomiting syndrome were negatively correlated with AHP, while psychiatric disorders and obstetrics and gynecology (OBGYN) disorders were positively correlated with AHP. The highest rate of diagnosis resulted from a combination of genetic and biochemical testing. Testing outside of an acute attack was not associated with a positive diagnosis.

**Conclusions:**

Patients with a history of OBGYN disorders and psychiatric disorders may be at increased risk of having AHP, yet there is a lack of involvement of these specialties in the diagnosis and care of AHP, in addition to a lack of studies investigating AHP in non-white populations potentially leading to reduced recognition of AHP.

## Introduction

Acute hepatic porphyria (AHP) is a group of four distinct metabolic diseases caused by genetic defects in the heme synthesis pathway [1]: The most common subtype, Acute Intermittent Porphyria (AIP), is caused by a variant in the *HMBS* gene, followed by Variegate Porphyria (VP), resulting from a *PPOX* gene variant and Hereditary Coproporphyria (HCP), which is caused by a variant in the *CPOX* gene. The rarest subtype is ALA dehydratase deficiency porphyria (ADP), caused by a variant in the *ALAD* gene [2]. They present similarly as episodic, severe neurovisceral abdominal pain that builds up over a few days and can be difficult to manage, frequently requiring high doses of narcotic pain medications in addition to hospitalization and supportive care. It is thought that attacks are directly related to an accumulation of the heme precursors porphobilinogen (PBG) and/or delta-aminolaevulinic acid (delta-ALA or d-ALA), which appear to have neurotoxic (among likely other pathogenic) properties, leading to long-term end-organ damage and comorbidities such as kidney and liver disease [3, 4] as well as higher rates of miscarriage, low birth weight, and infertility [5, 6] if the disease is left untreated. However, ongoing research also indicates that there are other mechanisms involved in the neurotoxic effects seen in these diseases [6–9], There are currently two approved therapies for AHP: Panhematin (Hemin), an acute phase therapy, and a recently FDA-approved RNA silencing therapy for prevention, Givosiran (Givlaari, Alnylam Pharmaceuticals, Cambridge MA, USA) [10]. Financial assistance programs (subject to country, region, and insurance restrictions) are available to reduce or eliminate the cost for those who are under or uninsured or otherwise socioeconomically disadvantaged.

In particular, the subtype AIP, with a currently diagnosed symptomatic population prevalence of 1:20,000 [2, 3, 11–15] shows a significant lack of studies in demographics other than European White (Caucasian) females. This despite a carrier prevalence as high as 1:1,500 [2] and being easily mistaken for any other cause of acute abdominal pain, including cholecystitis, appendicitis, pancreatitis, urinary tract disease [6, 16] and OBGYN related pathology [6, 17]. This leads to AIP patients frequently being misdiagnosed, especially in non-Whites and perhaps even males, who may present atypically. While it is commonly accepted that AIP appears more frequent in both carrier status and symptomatic prevalence in patients of Caucasian background [2], this assumption may be due to most AIP studies being conducted on Caucasian-background patients, against which future studies were then evaluated. Naturally, the common genetic variants found in the predominantly Nordic European populations of the largest contributor studies are not commonly found in other ethnicities, adding to the assumption that AIP affects predominantly patients of Caucasian heritage and patients of female sex, despite the AIP causing genetic variants being inherited equally among the sexes [2, 10]. There are multiple studies from other countries highlighting surprising symptomatic prevalence rates of AIP as high as 1.16% among other ethnicities, such as Northern India [17], where patients present primarily with Acute Flaccid Paralysis and neuropathies, and studies from Mexico including one showing a 0.67% symptomatic prevalence of AIP among the tested psychiatric populations [18].

Correct identification of the 4-disease group takes, on average, 10–15 years and often requires access to tertiary care, provider familiarity with disease, and testing [19], which typically takes a week or longer to result, often after the patient is discharged and lost to follow-up. Hence, AHP disproportionately affects the un- and underinsured socioeconomically disadvantaged patient populations, who are unable to return to clinic appointments due to lack of paid sick time, transportation, and health insurance coverage, placing them at greatest risk for remaining undiagnosed and developing long-term irreversible comorbidities due to lack of treatment.

During our most recent UCLA-UCSF joint study [20] aimed at reducing diagnostic delays in AHP using machine learning algorithms, we discovered that the current number of patients diagnosed with AHP within the UCLA Health patient population is 0.0034%, much lower than the symptomatic population prevalence estimate across the United States of 0.005% [3, 4], yielding an under-diagnosis rate of 32% in comparison to the general US population prevalence. Considering that UCLA Health is a tertiary care healthcare system, we should see a diagnosis rate that exceeds the estimated population symptomatic prevalence. Our study aims to suggest areas of targeted intervention to identify undiagnosed patient cohorts: patients who may present atypically, such as males and patients of other ethnicities, who often may go undiagnosed due to structural barriers to specialist healthcare access and/or logistical testing difficulties.

This study aims to evaluate a cohort of patients who have recently been evaluated for AHP (2019–2022) to investigate phenotypic differences and correlations in demographics and clinical presentations by AHP subtype that may assist providers in clinical decision-making to increase rates of AHP diagnosis specifically in non-White and atypically presenting patients in the future.

## Materials and methods

### Study population

This is a cohort study of a total of 45 patients at the University of Los Angeles California (UCLA) Health System who have undergone evaluation for AHP between 2019 and 2022. An initial data inquiry resulted in 153 patients with an International Statistical Classification of Diseases and Related Health Problems (ICD) code of any of the AHP subtypes. A manual chart review was then conducted to further classify patients into the AHP + cohort, the AHP- cohort or “other”, which was a cohort consisting of patients not utilized for this study due to either pending diagnostic testing or insufficient data in the chart. This is a cohort study using retrospective chart review of EMR data of patients diagnosed with any of the AHP subtypes except ALAD, from UCLA between 2019 and 2022. The authors are also currently analyzing cohorts from other institutions and will periodically publish updates with a larger sample size.

Inclusion criteria consisted of patients over 18 years of age with a true AHP diagnosis code for the AHP + cohort (specified as an ICD code for any of the AHP subtypes + either a confirmatory genetic test and/or confirmatory biochemistry) and a tentative AHP diagnosis code for the AHP- cohort (specified as patients over 18 years of age with an ICD code for any of the AHP subtypes + subsequent negative genetic testing and/or negative biochemistry when symptomatic and/or a satisfactory alternative diagnosis was found. 27 patients ultimately were diagnosed with AHP (AHP + cases) and 18 were ruled out for the disease (AHP- controls). Biochemistry included spot urine for ALA and PBG elevations above the normal limit per Quest labs 2019–2022 as well as urinary total porphyrins elevations above the normal limit per Mayo Clinic labs 2019–2022 (see tables for details).

Electronic Medical Record (EMR) data was collected from both groups for comparison and correlation analysis, including quantitative and qualitative information: demographics, clinical notes including past medical history, laboratory results including genetic variants if available, referral and diagnosis sources, comorbid conditions, family history, and medication lists. Through manual chart review, symptoms, comorbidities, clinical histories, lab results and health system touch points for diagnosis and referral journey were collected for each individual patient and prepared for comparisons in frequency within the AHP + and AHP- cohort as well as between the cohorts. Clinical data was included in this study if it was presented more than once among a cohort or subtype cohort.

AHP + patients were then grouped into AHP subtype cohorts: Acute Intermittent Porphyria (AIP/ *HMBS* variant), Variegate Porphyria (VP/*PPOX* variant), and Hereditary Coproporphyria (HCP/*CPOX* variant) to investigate phenotypic differences and commonalities in genotypes. There were no patients with the ALA Dehydratase Deficiency (ADP/*ALAD* variant) subtype in this cohort.

The following conditions were grouped together as potentially correlated comorbidities:

“OBGYN disorders”: vaginal itching/discomfort unspecified, spotting, irregular periods, ovarian cysts, dyspareunia, infertility, endometriosis, history of hysterectomy, history of miscarriage, cholestasis of pregnancy, premature delivery, fibroids, dysmenorrhea.

“Psychiatric disorders: Major Depressive Disorder, General Anxiety Disorder, Schizophrenia, Psychosis, Hallucinations, Attention Deficit and Hyperactivity Disorder.

“Urinary symptoms”: defined as discolored urine, urinary urgency, increased urinary frequency, burning/discomfort with urination without presence of UTI, cystitis without presence of infection.

### Data analysis

Both descriptive and inferential statistical analysis of the data was carried out. Fisher’s Exact Test was used to find the significant difference in different parameters and values. Statistical significance was established at a p-value of 0.05 or less with a 95% Confidence Interval. All statistical calculations were performed using SPSS (IBM Corp., Armonk, NY) version 29.0.0.

## Results

### Demographics (Table 1)

**Table 1:** Sociodemographic features on the basis of presence or absence of AHP

		AHP+, (*n* = 27) *n*, (percentage)	AHP– (*n* = 18) *n*, (percentage)	*p*-value
Gender	Male	5 (19)	5 (28)	0.489
	Female	22 (81)	13 (72)	
Age	Minimum	24	24	0.791
	Maximum	70	79	
	Mean (SD)	47 (16)	49.33 (19)	
Ethnicity	White	21 (78)	14 (78)	0.148
	Non-Hispanic	0	1 (6)	
	Asian	0	1 (6)	
	decline/unknown	0	1 (6)	
	Hispanic or Latino	4 (15)	0 (0)	
	other/Non-Hispanic	2 (7)	1 (6)	
Family History	Yes	13 (48)	2 (11)	< 0.001*
AHP subtype	AIP	18 (67)		
	VP	6 (22)		
	HCP	3 (11)		

AHP: Acute Hepatic Porphyria, AIP: Acute Intermittent Porphyria, HCP: Hereditary Coproporphyria, VP: Variegate Porphyria, n = total number of patients

*p < 0.05 is statistically significant

#### Age and gender

In the AHP + cohort, 81% were female, and 19% were male, among the AHP- cohort, 72% were female, while 28% were male. The average patient age was 49.7 years. There was no significant difference found in gender or age distribution between the positive and negative groups.

### Ethnicity

The majority (78%) of patients across both the positive and negative cohort were White, followed by 9% of non-White Hispanic or Latino, 4% non-Hispanic/black, 2% Asian and 2% were of unknown ethnic origin. While there were no statistically significant differences in ethnicity distribution between the positive and negative groups, all patients identifying as Hispanic or Latino were AHP+, whereas all AHP- patients identified as Asians.

#### Family history

In the AHP-positive group, 48% reported having a family history of AHP, while 11% in the AHP- group reported a positive family history, leading to a statistically significant difference (*p* < 0.001).

#### AHP subtypes

The most common AHP subtype was AIP (67%) for both females and males, followed byVP(22%), which was more common in females than males although not statistically significant due to small sample size, and lastly, HCP (11%) with an equal distribution among females and males. There were no patients with the ALADPorphyria subtype in this study.

### Referral and diagnostic journey

Table − 1 suppl (Supplementary Data) indicates that 22% of patients came to UCLA based on self-referral for evaluation of Porphyria, followed by 15% of patients being referred from their primary care provider (PCP) and 7% by Hematology/Oncology. Interestingly, one patient was referred by their local veterinarian and ultimately tested positive for AHP.

**Fig. 1:**
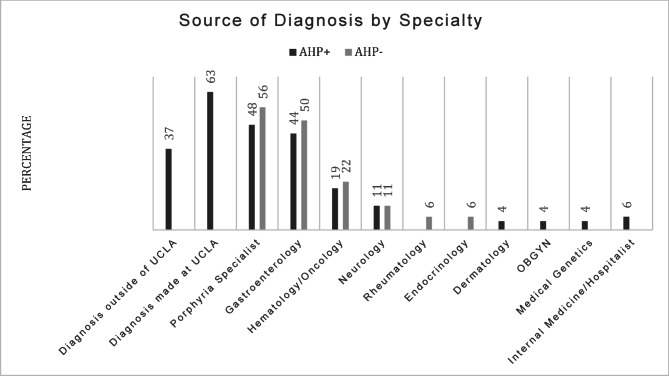
Source of diagnosis by specialty. AHP+:Acute Hepatic Porphyria Positive, AHP-:Acute Hepatic Porphyria negative, UCLA = University of California Los Angeles, OBGYN: Obstetrics and Gynecology

Figure 1 compares sources of diagnosis for AHP + and AHP- patients; 37% of AHP + patients had a prior diagnosis of AHP before coming to UCLA for further care, and 63% of patients were diagnosed at UCLA. The most common physician specialty to diagnose AHP was Gastroenterology (44%) followed by Hematology/Oncology (19%) and Neurology (11%). 1 patient was diagnosed through preconception genetic testing at their OBGYN. Additionally, 48% of AHP + patients had been evaluated by a Porphyria specialist, which can consist of any medical specialty. Evaluation of AHP- patients showed a similar pattern, with the majority being assessed by Gastroenterology, Hematology/Oncology, and Neurology. 56% of AHP- patients were formally assessed by a Porphyria specialist.

### Method of diagnosis

Table 2 provides an overview of all testing conducted in both AHP + and AHP- groups.

**Table 2:** Source of referral

Referred by	percentage	*n*
Self	22%	6
PCP to subspecialty: Rheumatology/Gastroenterology/Neurology	15%	4
Internal medicine/Hospitalist	11%	3
Genetics to Porphyria specialist	4%	1
Rheumatology to Genetics	4%	1
Pediatrics	4%	1
Hematology/Oncology to Porphyria Specialist	4%	1
General Dermatology to Specialist Dermatology	4%	1
Veterinarian to human Neurology	4%	1
Military cardiology to Hematology/Oncology	4%	1
Neurology to Porphyria Specialist	4%	1

PCP = Primary Care Provider, n = total number of patients

AHP was considered positive if the patient either had a positive genetic test for a known pathogenic variant in the genes *HMBS*, *CPOX*, *PPOX* and/or positive biochemistry Urinalysis (UA) in the absence of a genetic test (asymptomatic carriers were included in this study). Qualifying biochemistry included elevations of any one or combination of random urinary delta- aminolaevulinic acid (d-ALA) > 5.4 mg/g creatinine, urinary random Porphobilinogen (PBG) > 2 mg/g creatinine, 24-hour Total Porphyrins > 120 µg/L (144 nmol/L). Uroporphyrins > 2 mcg/dL (< 2.4 nmol/L), Coproporphyrins > 2 mcg/dL (< 30 nmol/L) are listed but did not determine AHP status alone without a confirmatory genetic test.

Whole Blood (WB) Porphyrins are mentioned with a normal cut-off value of < 80 mcg/dL but did not affect a patient’s positive or negative status for AHP for this study: In the AHP + group, 63% had WB porphyrins taken, while in the APH- group 44% had WB porphyrins taken. Among those who had WB porphyrins taken, 35% in the AHP + group and 50% in the AHP- group had elevated levels of porphyrins (> 80 mcg/dL,), indicating no significant difference (*p* = 0.667).

### Genetic testing

Genetic test results were available for 74% in the AHP + group and for 39% of patients in the AHP- group. Within the AHP + group that had genetic testing done, 48% had *HMBS* variants, 19% had *PPOX* variants and 7% had *CPOX* variants. All available variants were heterozygous, with 2 patients having the same *HMBS* variant (1 male and 1 female: heterozygosity c.517 C > T heterozygous, which is a known pathogenic association with autosomal dominant AIP).

### Biochemistry

In both the AHP + and AHP- groups, 89% in both the AHP + and AHP- group had UAresults available for biochemistry evaluation. Among the AHP + group with available UA, a total of 71% samples were positive, containing at least one elevated value of qualifying biochemistry (random urinary d-ALA, PBG, total Porphyrins, Uroporphyrins, Coproporphyrins with the latter two categories necessitating a positive genetic test to be considered positive for AHP). None of the individuals in the AHP- group had positive UA results except for 1 patient who had elevated Coproporphyria I values with negative ALA and PBG, later found to have a *FECH* (ferrochelatase) variant and therefore was considered negative for the AHP subtypes included in this study.

63% of UAs taken from both the AHP + and AHP- group contained both ALA and PBG values. Of the AHP + positive UAs, 53% had either ALA or PBG or both (if both were available) elevated, 7% had PBG elevated but ALA negative, 20% had negative results for PBG but positive results for ALA. Further, 17% of UA samples that were negative for both ALA and PBG were positive for either Total Porphyrins, Coproporphyrins and/or Uroporphyrin (but had a positive genetic test).

Of the 50% of the AHP + UAs taken during a symptomatic attack, 58% were positive. Among the other 50% of AHP + UAs taken outside of an acute attack, 50% were positive also.

In the AHP + group, 81% of patients were symptomatic at the time of evaluation (Table 4 shows a list of symptoms considered for the purposes of this study). 74% had a positive genetic test for any of the AHP subtypes, 56% of patients had a positive biochemistry. 30% of all AHP + patients were symptomatic with a positive genetic test but had negative or not available biochemistry. 41% of AHP + patients had both a positive genetic test as well as positive biochemistry. One male patient had a positive genetic test with positive biochemistry but presented atypically with a Neurodevelopmental Disorder.

### Differential diagnoses in AHP- patients

39% of AHP- patients who were tested had a negative genetic test with only 1 patient being ruled out for AHP based on the negative genetic test alone. 33% of patients were ruled out for AHP based on a negative UA alone, and 28% had both a negative genetic test and negative UA.

Table 2 suppl (supplementary data) provides an overview of the final diagnoses given to patients in the AHP- cohort:

### Treatment of AHP + patients

52% of patients received Hemin/Panhematin, and 14% of those reported it not being effective for abdominal pain, as it only provided relief for neurological pain and parasthesias. 71% of patients on Hemin/Panhematin eventually discontinued the treatment due to the lack of full symptom relief seen in the patients. 26% of patients receive Givosiran [13], with 1 patient stating intolerance in the form of intermittent nausea, headache and dizziness for 14 days post injection (Latino ethnicity, *HMBS* heterozygous c.77G >A, p. (Arg26His) and one person reporting no relief (*HMBS* heterozygous, unknown exact variant) until the dosage was increased based on body weight. 15% of patients received Dextrose solutions, 15% used opioid medications, and in equal percentages of 4% patients utilized over the counter (OTC) pain relief, intravenous Immunoglobulin (IVIG), Lupron, Chinese Herbs and Cannabinoid (CBD).

19% of patients were not undergoing any specific treatment for AHP at the time of the data collection phase (January 1, 2021 – December 31, 2022).

### Clinical presentation of AHP + and AHP- patients

Symptoms began for 19% of AHP + patients regardless of genotype and gender with a seizure (unknown trigger and without pre-existing seizure-related conditions per chart review), followed by skin blisters and/or rashes (11%), which were overall more common in the VP subtype (7%). For the *HMBS* subtype, 11% of females stated symptoms began with pregnancy, with 1 person terminating their pregnancy due to intolerable worsening symptoms and 1 person receiving twice weekly Panhematin infusions throughout her pregnancy, giving birth to a child with infantile pyknocytosis thought to be due to Panhematin use.

Other patients reported symptoms that began after an accident (4%) and surgery (4%).

45% of female patients reported symptom worsening with menses.

**Fig. 2:**
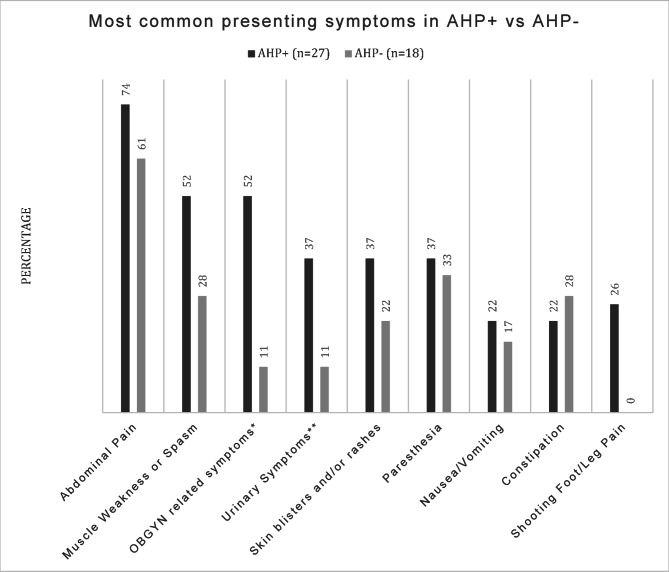
Symptom comparison between AHP + and AHP-. AHP + = Acute Hepatic Porphyria positive patients, AHP- = Acute Hepatic Porphyria negative patients. *OBGYN = Obstetrics and Gynecology, symptoms for this study included: vaginal itching/discomfort unspecified, spotting, irregular periods, ovarian cysts, dyspareunia, infertility, endometriosis, history of hysterectomy, miscarriage, cholestasis of pregnancy, premature delivery, fibroids, dysmenorrhea. **Urinary symptoms include discolored urine, urinary urgency, increased urinary frequency, burning/discomfort with urination without presence of UTI, cystitis without presence of infection

**Fig. 3:**
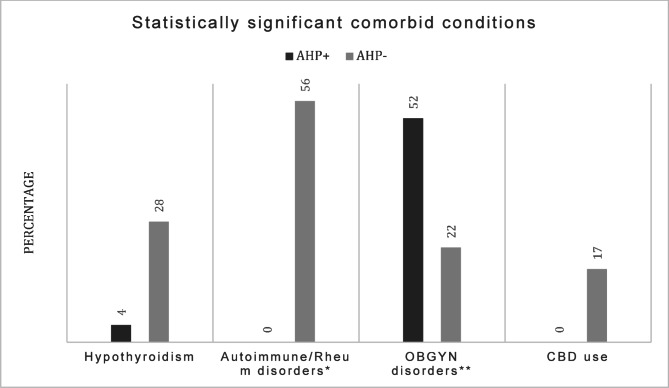
Statistically significant comorbid conditions correlated with presence or absence of Acute Hepatic Porphyria. AHP + = Acute Hepatic Porphyria positive patients, AHP- = Acute Hepatic Porphyria negative patients. CBD = Cannabidiol. *Autoimmune and rheumatological disorders include Hypothyroidism, Type 1 Diabetes, Fibromyalgia, Myasthenia Gravis. **OBGYN = Obstetrics and Gynecology, symptoms and disorders for this study included: vaginal itching/discomfort unspecified, spotting, irregular periods, ovarian cysts, dyspareunia, infertility, endometriosis, history of hysterectomy, miscarriage, cholestasis of pregnancy, premature delivery, fibroids, dysmenorrhea

Table 3; Fig. 2 show the presenting symptoms among AHP + and AHP- patients in our study, while Table 4; Fig. 3 present the results of a correlation analysis aimed at predicting likelihood of having AHP based on existing comorbidities and symptoms alone.

**Table 3:** Diagnostic methods

Ordered laboratory testing and symptomatic circumstances	AHP+		AHP-		*P* Value*
	percentage	Total (*n* = 27)	percentage	Total (*n* = 18)	
Genetic Testing for AHP (HMBS, CPOX, PPOX)	74%	20	39%	7	
Positive genetic test result for AHP (HMBS, CPOX, PPOX) of those tested	100%	20	0%	0	< 0.001*
Negative genetic test result for AHP for those tested	0%	0	100%	7	
Total Whole Blood Porphyrins taken	63%	17	44%	8	
WB porphyrins elevated:>80 mcg/dL	35%	6	50%	4	0.667
WB Porphyrins normal < 80 mcg/dL	65%	11	50%	4	
Total number of urinalysis (UA) taken	89%	24	89%	16	
Number of positive UA of those tested	71%	17	6%**	1**	< 0.001*
Urinalysis taken when symptomatic	50%	12	69%	11	
Urinalysis taken when not symptomatic	50%	12	31%	5	
Positive UA when symptomatic	58%	7	0%	0	
Negative UA when symptomatic	42%	5	100%	11	
Positive UA when not symptomatic	50%	6	0%	0	
Negative UA when not symptomatic	50%	6	100%	5	
**Urinalysis breakdown by compound**					
Number of UAs with both ALA and PBG available	63%	15	63%	10	
Fraction of positive UAs with either ALA or PBG or both if available elevated	53%	9	0%	0	
Fraction of positive UAs with both ALA and PBG negative but + for other compounds	24%	4	6%**	1**	
Of positive UAs with both ALA and PBG available, PBG- but ALA+	20%	3	0%	0	
Of positive UAS with both ALA and PBG available, PBG + but ALA-	7%	1	0%	0	
Fraction of positive UAs where ALA and PBG were not done or negative but other values were positive	24%	4	6%**	1**	
Positive UAs with Uroporphyrins and Coproporphyrins only, no ALA/PBG	6%	1	6%**	1**	
Positive UAs with no ALA/PBG but total Porphyrins	12%	2	0%	0	
Positive UAs with total Porphyrins, Uroporphyrins and Coproporphyrins but no ALA/PBG	6%	1	0%	0	

AHP = Acute Hepatic Porphyria, + = positive, - = negative, HMBS = hydroxymethylbilane synthase, CPOX = Coproporphyrinogen Oxidase, PPOX = protoporphyrinogen oxidase, WB = Whole Blood, UA = Urinalysis, ALA = Aminolevulinic acid dehydratase, PBG = Porphobilinogen

*p < 0.05 is statistically significant

**1 patient in the AHP- group had elevated Coproporphyrin I values with negative ALA and PBG, later found to have a FECH variant and therefore was considered negative for the AHP subtypes included in this study (AIP, VP, HCP, ADP only)

**Table 4:** Comparison of frequently presenting symptoms between AHP- and AHP + patients

Presenting Symptoms	AHP+, (*n* = 27), %	AHP-, (*n* = 18), %
Visual and vestibular disturbances	2 (7)	2 (11)
Urinary symptoms*	10 (37)	2 (11)
Skin blisters or rashes	10 (37)	4 (22)
Abdominal pain	20 (74)	11 (61)
Bloating	0 (0)	5 (28)
Diarrhea	0 (0)	5 (28)
Paresthesia	10 (37)	6 (33)
Muscle weakness or spasms	14 (52)	5 (28)
Dysphagia	0 (0)	2 (11)
Nausea/Vomiting	6 (22)	3 (17)
Constipation	6 (22)	5 (28)
Tachycardia/palpitations	3 (11)	1 (11)
Body aches and pains	0 (0)	4 (22)
fatigue/easy fatigability	0 (0)	4 (22)
Back pain	4 (15)	2 (11)
Stabbing foot/leg pain	7 (26)	0 (0)
OBGYN disorders and symptoms**	14 (52)	2 (11)
Non-AHP medication brings relief (including Amitriptyline, Antihistamine, Steroids and Proton Pump Inhibitors)	0 (0)	6 (33)
Multiple allergies (> 5) listed in chart	0 (0)	4 (22)
Symptoms triggered by food	0 (0)	5 (28)

AHP + = Acute Hepatic Porphyria positive, AHP- = Acute Hepatic Porphyria negative

*Urinary symptoms include discolored urine, urinary urgency, increased urinary frequency, burning/discomfort with urination without presence of UTI, cystitis without presence of infection

**OBGYN disorders and symptoms = Obstetrics and Gynecology, symptoms for this study included: vaginal itching/discomfort unspecified, spotting, irregular periods, ovarian cysts, dyspareunia, infertility, endometriosis, history of hysterectomy, miscarriage, cholestasis of pregnancy, premature delivery, fibroids, dysmenorrhea

Notably, abdominal pain was the most prevalent symptom in both the positive and negative group (74% and 61%), followed by muscle weakness and/or spasms (52% and 28%). OBGYN-related symptoms and disorders (see Methods section for details) were reported in 52% of AHP + patients compared to 22% of AHP- patients, showing a significant but weak positive correlation (correlation coefficient: 0.296, *p* = 0.048) with AHP. The presence of psychiatric disorders (see Methods section for included conditions) was also more prevalent in the AHP + group with 52% vs. 28% in the AHP- group, showing a positive correlation with AHP, however it did not reach statistical significance (*p* = 0.114).

In contrast, hypothyroidism exhibited a significant negative correlation with AHP (correlation coefficient: -0.347, *p* = 0.020, and the presence of autoimmune and rheumatological disorders (including Hypothyroidism, Type I Diabetes, Fibromyalgia and Myasthenia Gravis) in general showed a significant negative correlation with AHP (correlation coefficient: -0.580, *p* < 0.001). Cannabidiol use (CBD) with cyclic vomiting syndrome showed a statistically significant negative correlation with AHP (-0.327, *p* = 0.028).

### Genotype phenotype correlations

Table 5 highlights the distribution of symptoms and comorbid conditions among AHP patients based on their genotypes.

**Table 5:** Final diagnosis of AHP- patients

Final Diagnosis	*n*	percentage
Malignancy	4	22%
New diagnosis of IBS/functional GI	2	11%
Autoimmune disorders total*	5	28%
Mast cell activation syndrome	2	11%
Medication reaction	1	6%
Endometriosis	1	6%
cyclic vomiting syndrome	3	17%
Undiagnosed**	2	11%
CIDP	2	11%
Shy Drager syndrome	1	6%
EPP	1	6%
Seizure disorder unspecified	1	6%

AHP- = Acute Hepatic Porphyria negative, n = total number of patients, IBS = irritable bowel syndrome, functional GI = functional gastrointestinal disorder, CIDP = Chronic Inflammatory Demyelinating Polyneuropathy, EPP = Erythropoietic Protoporphyria

*Autoimmune Disorders: Mast cell activation syndrome, CIDP, Shy Drager Syndrome

**Entered into the Unknown Rare Disease Network for further evaluation

Patients with the *CPOX* genotype had the highest percentage (100%) of individuals experiencing urinary symptoms (for details see the Methods section), followed by those with the *HMBS* genotype (54%) and none of the patients with *PPOX* variants reported urinary symptoms, with a statistically significant difference of *p* = 0.019. The presence of skin blisters and rashes showed a different trend, with the *PPOX* genotype exhibiting the highest percentage (80%), followed by *CPOX* genotype (50%), and *HMBS* genotype (23%). However, these differences did not reach statistical significance (*p* = 0.076).

While psychiatric comorbidities were more prevalent in the *HMBS* genotype (62%) and the *CPOX* genotype (100%), the *PPOX* genotype had a markedly reduced prevalence of only 20%, however due to small sample size this difference was not statistically significant (*p* = 0.222).

### Differences in AHP presentation based on gender

Tables 6, 7 and 8 presents a comparison of sociodemographic, clinical characteristics (including symptoms and comorbidities), and laboratory measurements between females and males with AHP.

**Table 6:** Comorbid conditions and symptoms associated with presence or absence of AHP

		Significance value p*
Irritable Bowel Syndrome (IBS)	Correlation Coefficient	0.036
	Sig. (2-tailed)	0.813
	N	45
Hypothyroidism	Correlation Coefficient	− 0.347
	Sig. (2-tailed)	0.020*
	N	45
Autoimmune/Rheum Disorders**	Correlation Coefficient	− 0.580
	Sig. (2-tailed)	0.000*
	N	45
Psychiatric Disorders***	Correlation Coefficient	0.239
	Sig. (2-tailed)	0.114
	N	45
Major Depressive Disorder (MDD)	Correlation Coefficient	− 0.095
	Sig. (2-tailed)	0.535
	N	45
General Anxiety Disorder (GAD)	Correlation Coefficient	0.109
	Sig. (2-tailed)	0.476
	N	45
Neurodevelopmental Disorders including Autism	Correlation Coefficient	0.176
	Sig. (2-tailed)	0.247
	N	45
Schizophrenia/Psychosis	Correlation Coefficient	0.100
	Sig. (2-tailed)	0.513
	N	45
Neuropathy	Correlation Coefficient	− 0.025
	Sig. (2-tailed)	0.870
	N	45
Structural Disorders ****	Correlation Coefficient	0.024
	Sig. (2-tailed)	0.877
	N	45
History of Guillain-Barr-Syndrome (GBS)	Correlation Coefficient	0.036
	Sig. (2-tailed)	0.813
	N	45
History of Seizures	Correlation Coefficient	0.181
	Sig. (2-tailed)	0.233
	N	45
Postural Orthostatic Tachycardia Syndrome (POTS)	Correlation Coefficient	− 0.145
	Sig. (2-tailed)	0.340
	N	45
Hypertension (HTN)	Correlation Coefficient	0.185
	Sig. (2-tailed)	0.225
	N	45
Opioid Use Disorder	Correlation Coefficient	0.176
	Sig. (2-tailed)	0.247
	N	45
OBGYN Disorders*****	Correlation Coefficient	0.296
	Sig. (2-tailed)	0.048*
	N	45
History of Miscarriage	Correlation Coefficient	− 0.064
	Sig. (2-tailed)	0.677
	N	45
Endometriosis	Correlation Coefficient	− 0.064
	Sig. (2-tailed)	0.677
	N	45
Irregular Menses including Spotting	Correlation Coefficient	0.144
	Sig. (2-tailed)	0.344
	N	45
Infertility	Correlation Coefficient	0.176
	Sig. (2-tailed)	0.247
	N	45
Ovarian Cysts	Correlation Coefficient	0.255
	Sig. (2-tailed)	0.091
	N	45
History of Gastroesophageal Reflux Disease (GERD)	Correlation Coefficient	0.096
	Sig. (2-tailed)	0.532
	N	45
Cannabidiol (CBD) use	Correlation Coefficient	− 0.327
	Sig. (2-tailed)	0.028*
	N	45
Type 1 Diabetes	Correlation Coefficient	− 0.185
	Sig. (2-tailed)	0.225
	N	45
Attention Deficit and Hyperactivity Disorder (ADHD)	Correlation Coefficient	− 0.185
	Sig. (2-tailed)	0.225
	N	45

AHP + = Acute Hepatic Porphyria positive patients, AHP- = Acute Hepatic Porphyria negative patients

CBD = Cannabidiol

*p < 0.05, Significant

**Autoimmune and rheumatological disorders include Hypothyroidism, Type 1 Diabetes, Fibromyalgia, Myasthenia Gravis

***Psychiatric Disorders include Major Depressive Disorder, General Anxiety Disorder, Schizophrenia, Psychosis, Hallucinations, Attention Deficit and Hyperactivity Disorder

****Structural Disorders include joint pains, replacements and surgeries, tendinopathy, Osteoarthritis, trigger finger

*****OBGYN = Obstetrics and Gynecology, symptoms and disorders for this study included: vaginal itching/discomfort unspecified, spotting, irregular periods, ovarian cysts, dyspareunia, infertility, endometriosis, history of hysterectomy, miscarriage, cholestasis of pregnancy, premature delivery, fibroids, dysmenorrhea

**Table 7:** Differences in symptoms or comorbid conditions of AHP + patients based on genotypes

		Gene variants								
		Unknown		CPOX		HMBS		PPOX		*P* Value
		Count	Column %	Count	Column %	Count	Column %	Count	Column %	
Urinary symptoms**	No	6	86%	0	0%	6	46%	5	100%	0.019*
	Yes	1	14%	2	100%	7	54%	0	0%	
Skin blisters and/or rashes	No	6	86%	1	50%	10	77%	1	20%	0.076
	Yes	1	14%	1	50%	3	23%	4	80%	
History of seizures	No	6	86%	1	50%	10	77%	3	60%	0.560
	Yes	1	14%	1	50%	3	23%	2	40%	
Symptoms worse with Menses	No	2	29%	1	50%	6	46%	3	60%	0.959
	Yes	3	43%	1	50%	5	39%	1	20%	
	male	2	29%	0	0%	2	15%	1	20%	
OBGYN-related symptoms and disorders***	No	1	14%	0	0%	4	31%	3	60%	0.603
	Yes	4	57%	2	100%	7	54%	1	20%	
	male	2	29%	0	0%	2	15%	1	20%	
Psychiatric Disorders****	No	4	57%	0	0%	5	39%	4	80%	0.222
	Yes	3	43%	2	100%	8	62%	1	20%	
Major Depressive Disorder (MDD)	No	6	86%	1	50%	12	92%	4	80%	0.416
	Yes	1	14%	1	50%	1	8%	1	20%	
General Anxiety Disorder (GAD)	No	6	86%	1	50%	9	69%	4	80%	0.780
	Yes	1	14%	1	50%	4	31%	1	20%	
Hallucinations	No	5	71%	2	100%	12	92%	5	100%	0.427
	Yes	2	29%	0	0%	1	8%	0	0%	
Schizophrenia	No	7	100%	2	100%	12	92%	5	100%	1.000
	Yes	0	0%	0	0%	1	8%	0	0%	
Neurodevelopmental Disorders including Autism	No	7	100%	2	100%	11	85%	5	100%	0.741
	Yes	0	0%	0	0%	2	15%	0	0%	
Psychosis	No	6	86%	1	50%	13	100%	5	100%	0.100
	Yes	1	14%	1	50%	0	0%	0	0%	
Post traumatic Stress Disorder (PTSD)	No	7	100%	2	100%	11	85%	5	100%	0.741
	Yes	0	0%	0	0%	2	15%	0	0%	
Hypothyroidism	No	7	100%	2	100%	13	100%	4	80%	0.259
	Yes	0	0%	0	0%	0	0%	1	20%	
Hypertension (HTN)	No	3	43%	2	100%	9	69%	4	80%	0.514
	Yes	4	57%	0	0%	4	31%	1	20%	
Guillen-Barr-Syndrome (GBS)	No	7	100%	2	100%	11	85%	4	80%	0.658
	Yes	0	0%	0	0%	2	15%	1	20%	
History of Gastroesophageal Disease (GERD)	No	5	71%	2	100%	12	92%	5	100%	0.427
	Yes	2	29%	0	0%	1	8%	0	0%	
CBD use	No	6	86%	2	100%	10	77%	4	80%	1.000
	Yes	1	14%	0	0%	3	23%	1	20%	
History of Abdominal Surgeries	No	6	86%	2	100%	9	69%	5	100%	0.565
	Yes	1	14%	0	0%	4	31%	0	0%	

HMBS = hydroxymethylbilane synthase, CPOX = Coproporphyrinogen Oxidase, PPOX = protoporphyrinogen oxidase

AIP: Acute Intermittent Porphyria, HCP: Hereditary Coproporphyria, VP: Variegate Porphyria, N = total number of patients, CBD = Cannabidiol

*p < 0.05 is statistically significant, determined via Fischer’s Exact Test

**Urinary symptoms include discolored urine, urinary urgency, increased urinary frequency, burning/discomfort with urination without presence of UTI, cystitis without presence of infection

***OBGYN = Obstetrics and Gynecology, symptoms and disorders for this study included: vaginal itching/discomfort unspecified, spotting, irregular periods, ovarian cysts, dyspareunia, infertility, endometriosis, history of hysterectomy, miscarriage, cholestasis of pregnancy, premature delivery, fibroids, dysmenorrhea

***Psychiatric Disorders include Major Depressive Disorder, General Anxiety Disorder, Schizophrenia, Psychosis, Hallucinations, Attention Deficit and Hyperactivity Disorder

**Table 8:** Clinical presentation and presenting symptoms of AHP + vs. AHP- with gender breakdown

			Gender								*P* Value*
			Female				Male				
			*n*	Column %	M	SD	*n*	Column %	M	SD	
AHP subtype		AIP	15	68%			3	60%			0.773
		HCP	2	9%			1	20%			
		VP	5	23%			1	20%			
Gene variant		Unknown	5	23%			2	40%			0.876
		CPOX	2	9%			0	0%			
		HMBS	11	50%			2	40%			
		PPOX	4	18%			1	20%			
Age					46.2	15.7			50.4	20.7	0.612
Ethnicity		Egyptian	1	5%			0	0%			1.000
		Hispanic	3	14%			0	0%			
		Other, not Hispanic/Black	1	5%			0	0%			
		Peruvian	1	5%			0	0%			
		white	16	73%			5	100%			
Symptoms and Comorbidities	Urinary symptoms**	No	13	59%			4	80%			0.621
		Yes	9	41%			1	20%			
	Skin blisters and rashes	No	14	64%			4	80%			0.636
		Yes	8	36%			1	20%			
	History of Seizures	No	18	82%			2	40%			0.091
		Yes	4	18%			3	60%			
	Psychiatric disorders***	No	11	50%			2	40%			1.000
		Yes	11	50%			3	60%			
	Major Depressive Disorder	No	18	82%			5	100%			0.561
		Yes	4	18%			0	0%			
	Generalized Anxiety Disorder	No	15	68%			5	100%			0.283
		Yes	7	32%			0	0%			
	Hallucinations	No	20	91%			4	80%			0.474
		Yes	2	9%			1	20%			
	Schizophrenia	No	21	96%			5	100%			1.000
		Yes	1	5%			0	0%			
	Neurodevelopmental Disorders including Autism	No	22	100%			3	60%			0.028*
		Yes	0	0%			2	40%			
	Psychosis	No	20	91%			5	100%			1.000
		Yes	2	9%			0	0%			
	Post Traumatic Stress Disorder	No	20	91%			5	100%			1.000
		Yes	2	9.1%			0	0%			
	Hypothyroidism	No	21	96%			5	100%			1.000
		Yes	1	5%			0	0%			
	Hypertension	No	16	73%			2	40%			0.295
		Yes	6	27%			3	60%			
	History of Guillain-Barr Syndrome	No	19	86%			5	100%			0.605
		Yes	3	14%			0	0%			
	History of GERD	No	20	91%			4	80%			0.474
		Yes	2	9%			1	20%			
	CBD use	No	19	86%			3	60%			0.221
		Yes	3	14%			2	40%			
	History of abdominal surgeries	No	17	77%			5	100%			0.547
		Yes	5	23%			0	0%			

AHP + = Acute Hepatic Porphyria positive patients, AHP- = Acute Hepatic Porphyria negative patients

HMBS = hydroxymethylbilane synthase, CPOX = Coproporphyrinogen Oxidase, PPOX = protoporphyrinogen oxidase, AIP: Acute Intermittent Porphyria, HCP: Hereditary Coproporphyria, VP: Variegate Porphyria, n = total number of patients, GERD = Gastroesophageal Disease, CBD = Cannabidiol

*p < 0.05, Significant, determined via Fischer’s Exact Test and Independent Samples t-test

**OBGYN = Obstetrics and Gynecology, symptoms and disorders for this study included: vaginal itching/discomfort unspecified, spotting, irregular periods, ovarian cysts, dyspareunia, infertility, endometriosis, history of hysterectomy, miscarriage, cholestasis of pregnancy, premature delivery, fibroids, dysmenorrhea

***Urinary symptoms include discolored urine, urinary urgency, increased urinary frequency, burning/discomfort with urination without presence of UTI, cystitis without presence of infection

****Psychiatric Disorders include Major Depressive Disorder, General Anxiety Disorder, Schizophrenia, Psychosis, Hallucinations, Attention Deficit and Hyperactivity Disorder

Across both AHP + and AHP- cohorts, females constituted 68%, while males accounted for 32%. The distribution of the subtypes of AHP (AIP, HCP, VP) did not significantly differ between females and males (*p* = 0.773). The most common gene variant was *HMBS* for 50% of females and 40% of males. There were no statistically significant differences between females and males in clinical presentation (symptoms and/or comorbidities), except for the presence of neurodevelopmental disorders, which were present only in the male AHP + cohort (40% of males, *p* = 0.028).

## Discussion

The study aimed to expand upon existing clinical recommendations to reduce the diagnostic delay and improve rates of diagnosis of AHP in our diverse community. We investigated to what extent demographics, symptomatic presentation, and comorbidities play a role in predicting AHP and what combination of testing could lead to the highest rates of diagnosis. Finally, we attempted to lay the groundwork for future investigations into potential genotype-phenotype correlations and phenotypic presentation differences based on ethnicity to further enable precision testing and treatment in the future.

### Demographics – ethnicity and gender

Per the most recent population census conducted in California in 2022 [21], the largest ethnic group in Los Angeles County, the home of UCLA Health, is Hispanic or Latino (49%), followed by White (25%), Asian (16%) and Black or African American (9%) with 50% identifying as female and 34% identifying additionally as foreign persons/non-citizens or residents. There is a significant mismatch between the number of White patients (78%) evaluated for AHP at UCLA in comparison to any other ethnicity considering that Whites represent a minority of the population in Los Angeles County. Historically, it has always been assumed that AHP is more common in White/Caucasian and female patients [3, 20, 22], however there are several studies from other countries indicating a surprising symptomatic prevalence among non-White ethnicities [17, 18] with atypical presentations [18, 19] which is currently not reflected in the US-based AHP evaluation guidelines.

Additionally, at least the total number of evaluated patients from Hispanic and male demographics should be higher to more closely reflect the local population which is comprised of nearly half Hispanic/Latinos. However, in our study, only ~ 9% of patients evaluated for AHP were Hispanic and all of them tested positive for AHP and only 22% of the entire patient cohort evaluated for AHP were male and half of them tested positive for AHP. While it is possible that AHP is not as common in non-White ethnicities and males, it is interesting to note the large percentage of positive patients of the non-White (100%) and male patients tested (50%), in comparison to the positive patient percentage of the evaluated White patients. Analysis of potential differences between AHP + males and females also suggested that men may present atypically with less abdominal pain than women (60% prevalence vs. 77%) and statistically significantly more neurodevelopmental comorbidities than women regardless of the AHP subtype.

Ensuring increased testing of males and non-White patients may present an opportunity for intervention to increase diagnostic rates of AHP in our community and necessitates further studies in clinical and laboratory presentations of these specific patient subgroups to ensure updated presentation in our clinical AHP guidelines.

### Whom to test - symptomatic presentation and comorbidities

Hypothyroidism and Type 1 Diabetes were strongly negatively correlated with AHP, reducing the probability that the patient would test positive for AHP. There was also a trend of patients with a history of bariatric surgery presenting with AHP-like symptoms, however, none of them tested positive for AHP, but this warrants awareness. Lastly, CBD use with cyclic vomiting syndrome was also associated with the AHP-negative group, lowering the suspicion for AHP in those patients. Differential diagnoses to evaluate against in AHP-presenting patients may be malignancies of the GI system and the endocrine system, autoimmune and rheumatological conditions such as Systemic Mastocytosis, Connective Tissue Disorders, Dermatomyositis, and other neurological conditions such as CIDP.

#### The role of OBGYN in AHP

Considering the statistically significant increased prevalence of certain OBGYN-related disorders in the AHP + group in comparison to the AHP- group in our study, and a number of female AHP + patients reporting symptom onset or worsening with pregnancy to the extent of elective termination due to symptom intolerability and adverse outcomes for their newborn if they did carry to term, it is surprising that not more patients are being referred from and/or evaluated by OBGYN (only 1 patient). While a significant increase in especially pregnancy-related complications and comorbidities in AIP subtype patients particularly is well documented in other countries, there are no such studies in the US: Sweden has studied the correlations between infertility, miscarriage and AIP for decades [4], equally, studies from Greece [5], China [11], Bosnia [23] and India [24] have documented the increased prevalence of spontaneous abortions [5, 11], symptom worsening with pregnancy to the point of elective terminations [24], significant adverse maternal and infant outcomes in pregnancy of AIP patients such as maternal-infant mortality rates of up to 42%, low infant birth weight and maternal hypertension [5] and AIP mimicry of pre-eclampsia and eclampsia in patients with AIP [11]. As AHP is the great mimicker of so many other disorders, it is likely simply misdiagnosed within OBGYN, however considering the significantly increased adverse outcomes that are well documented, we might want to consider testing for AHP as part of the standard prenatal genetic screens and routine monthly urinalysis conducted at OBGYN offices throughout the course of pregnancy, at least for the duration of a study to assess prevalence.

### The role of psychiatry in AHP

Similarly to OBGYN, Psychiatry is an underrepresented field of referral and diagnosis source for AHP; in fact, not a single patient was referred to further care or diagnosed by a psychiatrist in our study, despite the high prevalence of psychiatric disorders in our study. The increased prevalence of psychiatric disorders and the mimicry of specifically AIP causing misdiagnosis of Schizophrenia, Psychosis, Hallucinations, Bipolar I, Major Depressive Disorder and General Anxiety Disorder has already been studied and is well documented [25], even in the US [26], confirming our results of a disproportionate prevalence of Hallucinations (especially in the HMBS/AIP subtype), Schizophrenia, Psychosis, MDD and GAD in our AHP + patient population.

Extending research and clinical involvement of AHP into the field of Psychiatry would likely lead to higher rates of AHP diagnosis and reducing misdiagnosis.

### Laboratory testing recommendations

The 2021 study by Karl Anderson et al. [22] discussed the current testing recommendations for AHP, preferring urinary PBG over ALA as the gold standard in significance, however it was suggested to review these recommendations every 2–3 years. In our patient cohort, the most common first line of testing was also urinary ALA and/or PBG, while Whole Blood (WB) Porphyrin testing is second in frequency, without showing any actual diagnostic ability to distinguish between AHP + and AHP- patients per our data from our patient cohort. Across both genders, if we only consider ALA and/or PBG then only 56% of patients would have tested positive for AHP; overlooking the 17% of AHP + patients who tested negative for ALA/PBG but were positive by symptoms and genetic testing. Furthermore, using only one value (ALA or PBG) would have only identified 17% of positive patients. Adding Total Urinary Porphyrins, Coproporphyrins and Uroporphyrins to the biochemistry testing increased our positive patient cohort from 56% to 74%, increasing the chance of diagnosis. The likelihood of an AHP patient testing positive based on urinary metabolites alone was 50% regardless of whether they were tested during an acute attack. Half of all AHP + patients who were tested during an attack tested negative for urinary metabolites while half of patients tested outside of an attack also tested positive for urinary metabolites. The rationale for including ‘asymptomatic carriers’ was based on the hypothesis of whether patients who presented clinical symptoms had any positive biochemical testing (ALA/PBG testing), and we found that to be the case. Thus, the authors strongly feel that this aspect should be further investigated.

If we had only determined AHP status by available genetic testing, we would have captured 74% of all AHP + patients, higher than standard biochemistry (urinary ALA/PBG, 56%) alone. However, genetic testing still carries a stigma with many populations [6]. Variants of Unknown Significance (VUS) may result in a patient not receiving the correct diagnosis [27, 28], which plagues the diagnosis of many rare diseases, including AHP; One of the most challenging things in clinical practice is working with a patient who presents with a genetic test result in hand but who does not have a typical clinical or biochemical metabolite trail to cement the diagnosis. Even among patients who have a known pathogenic variant in *HMBS*, the gene associated with AIP, not all of them experience an acute pain attack, these are known as latent porphyrics and their natural history remains poorly studied. One potential advantage of adding a targeted genetic screen for patients who present for a Porphyria evaluation may be to identify such latent porphyrics and could add substantially to our understanding of the natural history of Porphyrias. However, it is advisable to undertake this only in the setting of an academic research protocol. There are clearly environmental and physiologic factors–dehydration, starvation, anemia, certain medications—that impact whether a patient with a bona fide variant in an AHP gene will develop an acute attack [1, 27]. Given that clinical penetrance of individual genetic variants can be quite variable [1, 27], it is highly likely that there are other, unidentified modifier genes and pathways. In our experience at UCLA, only about 1 in 30 patients who come for a Porphyria evaluation actually end up getting a Porphyria diagnosis. Focusing on both provider and patient education about AHP should help sharpen diagnostic accuracy, improve clinical care and alleviate patient anxieties. Our cohort suggests that outreach to practitioners in the fields of mental health and obstetrics/gynecology may bear the most fruit and give them a resource of where to turn if they think about AHP but do not know what to do next. Providers also need to have a basic understanding of how to interpret ‘positive’ urine metabolite results. AHP is ruled in when urine metabolites that are elevated 10–100 times during a time of acute symptoms, whereas non-specific elevations of only two to three-fold are likely the result of dietary, processing, or other environmental factors. *Normal or trivially elevated urine metabolites during an acute pain attack rules out AHP however acute pain attacks are subjective and the patient should be tested frequently*,* not just once.* We typically give these patients and asymptomatic patients with elevated biochemistry (latent prophyrics) a prescription for appropriate urine tests to keep in their purse or wallet so that, in the future, if they present to a healthcare facility with symptoms suggestive of AHP, they can give the provider the correct tests to order. We also encourage the patients to invite their acute care provider to contact us to discuss the case, further testing, and supportive care.

### Treatment recommendations

The majority of patients are still being treated with Panhematin [6, 22], even in our cohort, however among our patients this treatment appears to be effective primarily for acute relief of neurological symptoms and improvement in biochemistry, with limited or no effect on abdominal pain. Further, it appears to lose its efficacy over time altogether.

A surprising number of patients (15%) are still being treated with Dextrose, which has led to significant self-reported weight gain in the dextrose-treated patients in our cohort.

Only about a quarter of our patients are treated with Givosiran, most likely due to difficulties in providers being aware of the treatment, the prior authorization process required by insurance, and the lack of knowledge that financial assistance programs (available in the US at present, and subject to country, region, and insurance plan restrictions) may be available for patients who are under or uninsured. It is also not yet known if Givosiran will work long term without losing efficacy, and if it is equally effective for all genotypes and ethnicities; further studies are needed. In our patient cohort, only 1 patient was unable to tolerate Givosiran (HMBS, Latino).

There also remains the question whether to treat asymptomatic AHP + patients. It is unknown at this time to what extent, if at all, clinical presentation is correlated with developing subsequent comorbidities known to affect AHP + patients, such as liver cancer. Long-term studies following asymptomatic carriers’ health outcomes are needed. Nevertheless, lifestyle adjustments, management of porphyrinogenic medications, and liver ultrasound in asymptomatic patients > 50 years age might be helpful strategies in treatment of this cohort of patients.

### Strengths and weaknesses

Patients were excluded from this study if insufficient EMR data was present leading to an inability to determine the true diagnosis of the patient; this could potentially mean that patients were left out of the study who in fact did have AHP or have been ruled out at another health center. Similarly, information was collected from both the standardized EMR documentation fields as well as the qualitative free note sections in the EMR, which is subject to the individual interpretation of the original recording physician, which played a role in determining whether a patient was suffering from an acute AHP attack at the time of the urinalysis. This in-depth manual chart review enabled capture of subjective qualitative data including attached pdfs from other health institutions enriched the details of this study, giving us a greater in depth look into Acute Hepatic Porphyria with potential to find new patterns that we otherwise would have not discovered. We have analyzed a very small cohort and merely observe trends or interesting individual cases. As we amass more data from additional centers, we may be able to draw statistical conclusions. In this manuscript we want to highlight that perhaps we are underdiagnosing certain populations based on previous assumptions made, such as that this disease is more frequently occurring in white/caucasian females traditionally. If we challenge these original thoughts, and they may very well be true, we should approach this and future cohort analyses with fresh eyes and consider that perhaps this disease is not as rare as we thought in non-caucasian/white female patients. We hope that with increased numbers in future cohorts we will be able to uncover additional, previously unknown patterns in this disorder for which there is not yet any data we can point to as supporting evidence.

Despite the small sample size, and therefore increased likelihood of Type II error, we hope this study can lay the groundwork for future more detailed investigations of phenotype-genotype correlations as well as clinical presentations in non-white and non-female patients to overall improve the rate of diagnosis of this disease, leading to earlier treatment and reducing unnecessary suffering and collateral comorbidities caused by AHP, especially in traditionally underserved patient populations.

### Summary of recommendations for clinical practice

#### Whom to test?

- AHP may present differently across age groups, sexes and ethnicities.
- Hispanic patients are equally as likely to suffer from AHP as White patients.
- Male patients may present atypically without classic abdominal pain, but with greater frequency of neurological and neurodevelopmental disorders including Autism.
- Patients with the following conditions may be less likely to have AHP:
  - Hypothyroidism
  - T1DM
  - History of bariatric surgery
  - History of CBD use with cyclic vomiting syndrome

- Patients with obstetric/gynecologic complaints may be more likely to have AHP and suffer from greater morbidities during pregnancy which warrants greater involvement of obstetricians/gynecologists in the diagnosis and care of AHP patients.
  - Vaginal itching/discomfort unspecified
  - Spotting or irregular periods
  - Ovarian cysts
  - Dyspareunia
  - Infertility
  - Endometriosis
  - History of hysterectomy, miscarriage, cholestasis of pregnancy, premature delivery, fibroids, dysmenorrhea
  - Symptoms that start with pregnancy
- Patients with mental health conditions may be more likely to have AHP, which warrants greater involvement of mental health providers in the diagnosis and care of AHP patients.
  - Major Depressive Disorder
  - General Anxiety Disorder
  - Schizophrenia
  - Psychosis
  - Hallucinations
  - Attention Deficit and Hyperactivity Disorder
  - Neurodevelopmental Disorders such as Autism, especially in males,

### How to test

- For the highest probability of a true positive test, include ideally both genetic screening as well as biochemical testing normalized to creatinine consisting of urinary ALA, PBG, Total Porphyrins, Coproporphyrins and Uroporphyrins regardless of the subtype of AHP suspected and irrespective of symptomatic presentation of the patient at the time of sample collection.
- Whole Blood Porphyrin testing has shown no ability to distinguish between our negative and positive patients.

### How to treat

- Avoidance of environmental and / or physiologic triggers (dehydration, starvation, anemia, and certain potentially Porphyria-triggering medications).
- IV dextrose to abort acute mild attacks can be very effective in the emergent care settings.
- Panhematin/Hemin is a commonly used acute phase treatment, however its limited availability in non-tertiary care settings and intricacies in its administration adds access difficulty. Whether tachyphylaxis to hemin might occur for some AHP patients in the long-term needs to be further investigated. The authors currently only believe that the penetrance is low and that it might be much higher if we have testing more readily available for symptomatic patients and traditionally underserved patients or atypical patient populations we did not historically test for this disease (i.e. nonwhite non-females).
- Givosiran (Givlaari) is a new siRNA-based treatment that effectively keeps AHP patients from experiencing further attacks of abdominal pain and avoids the need for hospitalization. Both Givosiran and hemin are very expensive and should only be used in consultation with a practitioner who has experience; financial assistance is available through the manufacturer Alnylam for patients who are unable to obtain coverage for the medication.
- The benefit of treating asymptomatic AHP + patients is unknown but considering the increased prevalence of certain comorbidities in the AHP + group such as OBGYN and psychiatric disorders, further studies are warranted to investigate this topic. The safety profiles for current treatment options are well established and this may warrant a low threshold to initiate treatment based on provider discretion after risk-benefit analysis. In addition, there should be consideration for open counseling on lifestyle choices, porphyrinogenic medications, and possibly liver ultrasound for the susceptible women in the age group > 50 years.

## Conclusions

This study highlights the critical need for improved diagnostic approaches and increased awareness of Acute Hepatic Porphyria (AHP) among diverse patient populations. The significant underdiagnosis in non-White and male patients suggests that current clinical guidelines and diagnostic practices may be insufficiently inclusive of these groups. Our findings advocate for broader and more inclusive testing criteria, incorporating genetic and biochemical tests beyond traditional urinary metabolites to enhance diagnostic accuracy. This does not undermine the significance for the awareness of measuring urine ALA/PBG during an episode of the disease that may still be relevant. We have observed trends of specific disorders that may be more common as potential comorbidities in patients with AHP. However due to the small sample size it is unclear if there is a pathophysiological reason or if these patients are simply seeking care more frequently and thus receive a broader workup to include the discovery of their AHP.

Additionally, increased education and outreach efforts targeted at healthcare providers in fields such as psychiatry and obstetrics/gynecology will further improve diagnostic rates. Future research should focus on genotype-phenotype correlations and the development of tailored treatment strategies to address the unique needs of diverse patient demographics. By addressing these gaps, we can move towards more equitable healthcare outcomes and improve the quality of life for all individuals affected by AHP.

## Data Availability

The authors state that it is not possible to publish all original source data from which this manuscript resulted due to privacy protection for our patient participants. In rare disease it is much easier to identify patients based on data points simply due to the fact that there are not many patients diagnosed with these diseases to begin with. This data can only be provided upon reasonable request to the corresponding author.

## Abbreviations

ALA: δ-Aminolaevulinic Acid
ALAD: ALA Dehydratase Deficiency
ADP: ALA Dehydratase Porphyria
AHP: Acute Hepatic Porphyria
CBD: Cannabinoid
CPOX: Coproporphyrinogen Oxidase
EMR: Electronic Medical Record
FECH: Ferrochelatase
HCP: Hereditary Coproporphyria
HMBS: Hydroxymethylbilane Synthase
ICD: International Statistical Classification of Diseases and Related Health Problems
IVIG: Intravenous Immunoglobulin
OBGYN: Obstetrics and Gynecology
PBG: Porphobilinogen
PPOX: Protoporphyrinogen Oxidase
RNA: Ribonucleic Acid
siRNA: Small Interfering RNA
T1DM: Type 1 Diabetes
UCLA: University of California Los Angeles
VP: Variegate Porphyria
